# Phytochemical Investigation of Bioactive Compounds from White Kidney Beans (Fruits of *Phaseolus multiflorus* var. *Albus*): Identification of Denatonium with Osteogenesis-Inducing Effect

**DOI:** 10.3390/plants10102205

**Published:** 2021-10-17

**Authors:** Yong Hoon Lee, Joo-Hyun Hong, Kun Hee Park, Seon-Hee Kim, Jin-Chul Kim, Do Hoon Kim, Yu Hwa Park, Kye Wan Lee, Jung Kyu Kim, Ki Hyun Kim

**Affiliations:** 1 School of Pharmacy, Sungkyunkwan University, Suwon 16419, Korea; yhl2090@naver.com (Y.H.L.); ehong@skku.edu (J.-H.H.); 2 Department of Food Science and Biotechnology, Sungkyunkwan University, Suwon 16419, Korea; soske@skku.edu; 3 Sungkyun Biotech Co., Ltd., Suwon 16419, Korea; seonhee31@gmail.com; 4 Natural Product Informatics Research Center, KIST Gangneung Institute of Natural Products, Gangneung 25451, Korea; jckim@kist.re.kr; 5 R&D Center, Dongkook Pharm. Co., Ltd., Suwon 16229, Korea; kdh2@dkpharm.co.kr (D.H.K.); pyh@dkpharm.co.kr (Y.H.P.); lkw1@dkpharm.co.kr (K.W.L.); 6 School of Chemical Engineering, Sungkyunkwan University, Suwon 16419, Korea

**Keywords:** *Phaseolus multiflorus* var. *albus*, Leguminosae, white kidney bean, denatonium, osteogenesis

## Abstract

*Phaseolus multiflorus* var. *albus* (Leguminosae), commonly known as “white kidney bean”, is a twining perennial vine whose fruit has been used as a popular food worldwide owing to its high nutritional content, in terms of proteins, carbohydrates, fats, and vitamins. As part of our ongoing study to investigate novel bioactive components from various natural resources, a phytochemical investigation of the extract of *P. multiflorus* var. *albus* fruits resulted in the isolation of three phenolic compounds (**1–3**) and one dipeptide (**4**). The chemical structures of the compounds (**1–4**) were determined through 1D and 2D nuclear magnetic resonance spectroscopy and high-resolution-liquid chromatography–mass spectrometry; they were identified as denatonium (**1**), *trans*-ferulic acid ethyl ester (**2**), eugenin (**3**), and α-L-glutamyl-L-Leucine (**4**). Intriguingly, denatonium (**1**) is known to be the most bitter chemical compound. To the best of our knowledge, denatonium (**1**) was identified from natural sources for the first time, and compounds **2–4** were reported for the first time from *P. multiflorus* var. *albus* in this study; however, compound **2** turned out to be an artifact produced by an extraction with ethanol. The isolated compounds **1–4** were tested for their regulatory effects on the differentiation between osteogenesis and adipogenesis of mesenchymal stem cells (MSCs). Compound **4** slightly suppressed the adipogenic differentiation of MSCs, and compounds **1** and **4** stimulated osteogenic differentiation, unlike the negative control. These findings provide experimental evidence that compounds **1** and **4** may induce the osteogenesis of MSCs and activate bone formation.

## 1. Introduction

*Phaseolus multiflorus* var. *albus*, also known as white kidney bean, is a twining perennial vine belonging to the family Leguminosae. It has been cultivated for many purposes worldwide, especially in China [1]. Historically, leguminous plants have been popular worldwide because they are abundant in proteins (16–33%), minerals, carbohydrates, fats, vitamins, and phytoestrogens [1,2]. Pharmacological studies of plants belonging to the genus *Phaseolus* have reported that its extracts exhibit various therapeutic properties, including antidiabetic [1,2], antiobesogenic [3], antiproliferative [4], antioxidant, and gastroprotective activities [5]. In addition, previous studies on plants belonging to the genus *Phaseolus* have shown that their nutritional contents possess insecticidal, immunomodulatory, antitumor, and antifungal activities [4,5]. Previous biological studies on *P. multiflorus* var. *albus* reported that white kidney bean (*P. multiflorus* var. *albus*) treatments induced glucose reduction and weight loss in a diabetes-induced mouse model compared to the control group [6]. In this context, many clinical trials on the antiobesity effect of *P. multiflorus* var. *albus* have revealed that the ingestion of white kidney beans induces weight loss and waist circumference reduction [7]. In a recent study, *P. multiflorus* var. *albus* extract showed dose-dependent inhibitory activity against α-amylase [8]. Despite the health benefits of *P. multiflorus* var. *albus* extract, few studies have been carried out on its chemical constituents. Only a few previous phytochemical investigations of *P. multiflorus* have shown the presence of gibberellins A_1_, A_5_, A_6_, A_8_, and phaseic acid as plant hormones with growth-promoting activity [9,10,11,12].

Therefore, as part of our ongoing study to investigate novel bioactive components from various natural resources [13,14,15,16,17,18,19], we investigated the potential bioactive components from the extract of *P. multiflorus* var. *albus* fruits. In the current study, the phytochemical constituents of the ethanolic extract of *P. multiflorus* var. *albus* fruits were isolated, resulting in the isolation of three phenolic compounds (**1–3**) and one dipeptide (**4**). The chemical structures of compounds (**1–4**) were clearly elucidated through 1D and 2D nuclear magnetic resonance (NMR) spectroscopic data and high-resolution electrospray ionization (HR-ESI) liquid chromatography–mass spectrometry (LC/MS) analyses. In addition, the isolated compounds, **1–4,** were tested for their regulatory effects on the differentiation between osteogenesis and adipogenesis of mesenchymal stem cells (MSCs). Herein, we report the isolation and structural determination of the isolated compounds **1–4** as well as their regulatory effects on the differentiation of MSCs.

## 2. Results and Discussion

### 2.1. Isolation of Compounds

**Figure 1:**
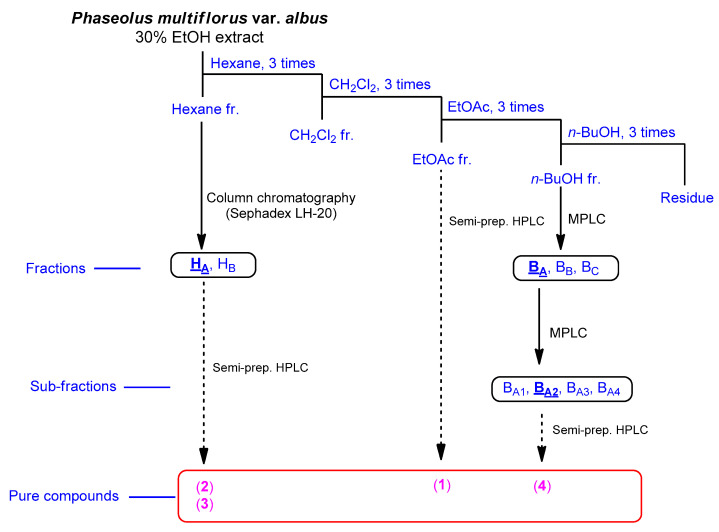
Separation scheme of compounds **1–4**.

**Figure 2:**
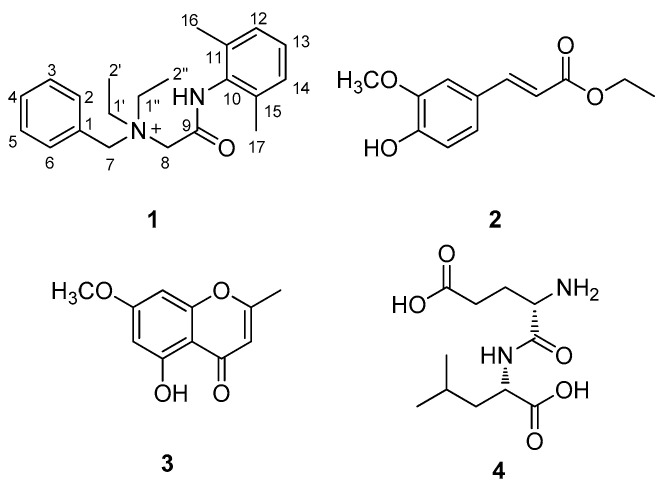
Chemical structures of compounds **1–4**.

The fruits of *P. multiflorus* var. *albus* were extracted with 30% ethanol/H_2_O. The resultant extract was suspended for solvent partitioning in water and then fractionated with four solvents, which afforded hexane-soluble (31.4 mg), dichloromethane-soluble (195.7 mg), ethyl acetate-soluble (49.3 mg), and *n*-butanol-soluble (2.4 g) fractions. The LC/MS-based analysis combined with our in-house built UV library and thin-layer chromatography (TLC) analysis of the solvent-partitioned fractions suggested that the BuOH-soluble fraction contained the majority of the organic acid derivatives. A phytochemical investigation of the solvent-partitioned fractions was conducted under monitoring by TLC and LC/MS-based analysis using repeated column chromatography with silica gel 60, RP-C_18_ silica gel, and Sephadex LH-20, and high-performance liquid chromatography (HPLC) (Figure 1). The final semi-preparative HPLC separation afforded a phenolic compound (**1**) from the EA-soluble fraction, two phenolic compounds (**2** and **3**) from the hexane-soluble fraction, and one dipeptide (**4**) from the *n*-butanol-soluble fraction (Figure 2).

### 2.2. Elucidation of Compound Structures

**Figure 3:**
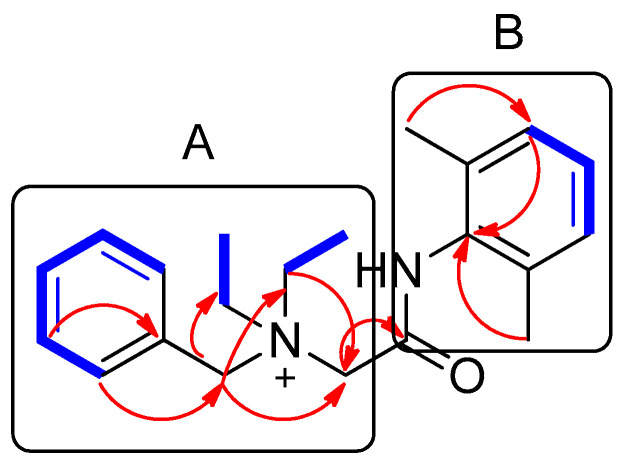
Key ^1^H-^1^H COSY (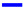) and HMBC (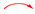) correlations of compound **1**. A and B represent two partial structures.

Compound **1** was isolated as a white amorphous powder. The molecular formula was deduced to be C_21_H_29_N_2_O^+^ from the molecular ion peak [M]^+^ at *m*/*z* 325.2282 (calculated for C_21_H_29_N_2_O^+^, 325.2274) in the positive-ion mode of HRESIMS (Figure S1). The ^1^H NMR spectrum of **1** (Table 1, Figure S2) displayed the presence of the characteristic signals of two aromatic proton sets of a monosubstituted aromatic ring at *δ*_H_ 7.64 (2H, t, *J* = 7.5 Hz), *δ*_H_ 7.62 (1H, t, *J* = 7.5 Hz), and *δ*_H_ 7.58 (2H, t, *J* = 7.5 Hz); 1,2,6-trisubstituted aromatic ring at *δ*_H_ 7.18 (1H, t, *J* = 7.0 Hz) and *δ*_H_ 7.17 (2H, d, *J* = 7.0 Hz); two pairs of relatively deshielded methylene groups at *δ*_H_ 4.94 (2H, s) and *δ*_H_ 4.16 (2H, s); two symmetric methyl groups at *δ*_H_ 2.30 (6H, s); and another two pairs of ethyl groups at *δ*_H_ 3.67 (4H, m) and *δ*_H_ 1.56 (6H, t, *J* = 7.5 Hz). The ^13^C NMR data of **1** (Table 1, Figure S3), assigned with the aid of the HSQC (Figure S5) and HMBC experiments (Figure S6)confirmed 21 carbon signals composed of four methyl groups at *δ*_C_ 8.4 (2 × C) and *δ*_C_ 18.7 (2 × C); four methylene carbons at *δ*_C_ 54.9 (2 × C), *δ*_C_ 55.7, and *δ*_C_ 63.4; 12 aromatic carbons (*δ*_C_ 128.7, 129.1, 129.5 (2 × C), 130.7 (2 × C), 132.3, 134.1 (2 × C), 134.2, 136.7 (2 × C)); and a carbonyl carbon at *δ*_C_ 164.1. The partial structures of **1** were determined by 2D NMR experiments (^1^H-^1^H COSY and HMBC). The gross structure of **1** was finally elucidated by the characteristic NMR signals, and its molecular formula (C_21_H_29_N_2_O^+^) was confirmed by HRESIMS. The ^1^H-^1^H COSY correlations (Figure S4) between H-2/H-3/H-4/H-5/H-6 as well as the HMBC correlations of H-2(H-6)/C-7 (*δ*_C_ 63.4) and H_2_-7/C-1, C-2, and C-3 verified the presence of benzyl functionality (Figure 3). Furthermore, the methylene of H_2_-7 showed HMBC correlations with three other carbons: C-8 (*δ*_C_ 55.7), C-1′ (*δ*_C_ 54.9), and C-1″ (*δ*_C_ 54.9) (Figure 3), providing evidence that a quaternary atom linking C-7, C-8, C-1′, and C-1″ is present. Based on the ^1^H-^1^H COSY spectrum of H_2_-1′/H_2_-2′ and H_2_-1″/H_2_-2″ and the HMBC correlations of H_2_-1′/H_2_-1″ with C-2′/C-2″, along with their symmetric NMR signals, the two ethyl units were assigned and confirmed to be attached to the quaternary atom by the HMBC correlations of H_2_-1′/H_2_-1″ with C-7 and C-8. The relatively deshielded methylene carbon NMR signals of C-7, C-8, C-1′, and C-1″ and the markedly diminished intensity of the carbon NMR signals observed for C-1′, and C-1″ at *δ*_C_ 54.9 confirmed that the quaternary atom linking them could be a quaternary ammonium cation, which finally led to the partial structure of A (Figure 3). Another spin system was observed as a cross-peak between H-12/H-13/H-14 in the ^1^H-^1^H COSY spectrum, representing the 1,2,6-trisubstituted aromatic ring, which was assigned to the 2,6-dimethylated benzene as the partial structure of B by the HMBC correlations of H-12(H-14)/C-10, H-13/C-11(C-15), C-16(C-17)/C-10, C-11, and C-12 (Figure 3). Finally, the connectivity through the amide bond between the two partial structures of A and B was suggested by the C=O and NH moieties remaining from the molecular formula (C_21_H_29_N_2_O^+^) of **1**, the detected HMBC correlation of H_2_-8/C-9 (*δ*_C_ 164.1), and the characteristic ^13^C chemical shifts of C-8 (*δ*_C_ 55.7) and C-10 (*δ*_C_ 134.2), although the key HMBC correlation between C-9 and C-10 was missing in **1** due to the absence of protons. Accordingly, the complete structure of **1** was established, as shown in Figure 1, and it was identified to be denatonium.

The structures of the known compounds (Figure 1) were determined to be *trans*-ferulic acid ethyl ester (**2**) [20], eugenin (**3**) [21], and α-L-glutamyl-L-Leucine (**4**) [22] by comparing their NMR spectroscopic data with those previously reported in the literature and MS data obtained from the LC/MS analysis. To the best of our knowledge, denatonium (**1**) was identified from a natural source for the first time, and compounds **2–4** were reported for the first time from *P. multiflorus* var. *albus* in this study. In natural product chemistry, it is important to take the necessary precautions during the isolation work in order to minimize the possibility of unexpected artifact isolation [23]. To verify whether compounds **1–4** were genuine natural compounds or artifacts, *P. multiflorus* var. *albus* was extracted with 80% methanol (*v*/*v*) for 10 h, and the resultant methanolic extract was subjected to LC/MS analysis. As a result, there was no peak with a molecular ion corresponding to compound **2** in the methanolic extract, whereas compounds **1**, **3**, and **4** were detected, suggesting that compound **2** was an artifact produced by the extraction with ethanol. In addition, to confirm that the isolated denatonium (**1**) is a natural compound, the methanol used for extraction was analyzed using ultra-performance liquid chromatography (UPLC) quadrupole time-of-flight (Q-TOF) high-resolution (HR)-MS because methanol can possess denatonium as a component. As a result, there was no detected peak for denatonium in the methanol solvent that we used for extraction (Figure S7), suggesting that the methanol used does not contain denatonium and the isolated denatonium can be a genuine natural compound.

Intriguingly, denatonium (benzyl-[2-(2,6-dimethylanilino)-2-oxoethyl]-diethylazanium), which is odorless and chemically stable, was found during research on local anesthetics in 1958 [24]. Since then, it has been widely used in various industries, such as cosmetics, pharmaceuticals, and material industries [25]. Interestingly, its bitterness and aversive taste have served to prevent young children from swallowing small household items, including toys and game packs, which has allowed denatonium to be widely employed in many household items. Denatonium is known to be one of the most bitter chemical compounds; thus, it was nominated in the Guinness Book of World Records as one of the most bitter compounds that people can use [26].

### 2.3. Evaluation of the Biological Activities of Compounds ***1***–***4***

**Figure 4:**
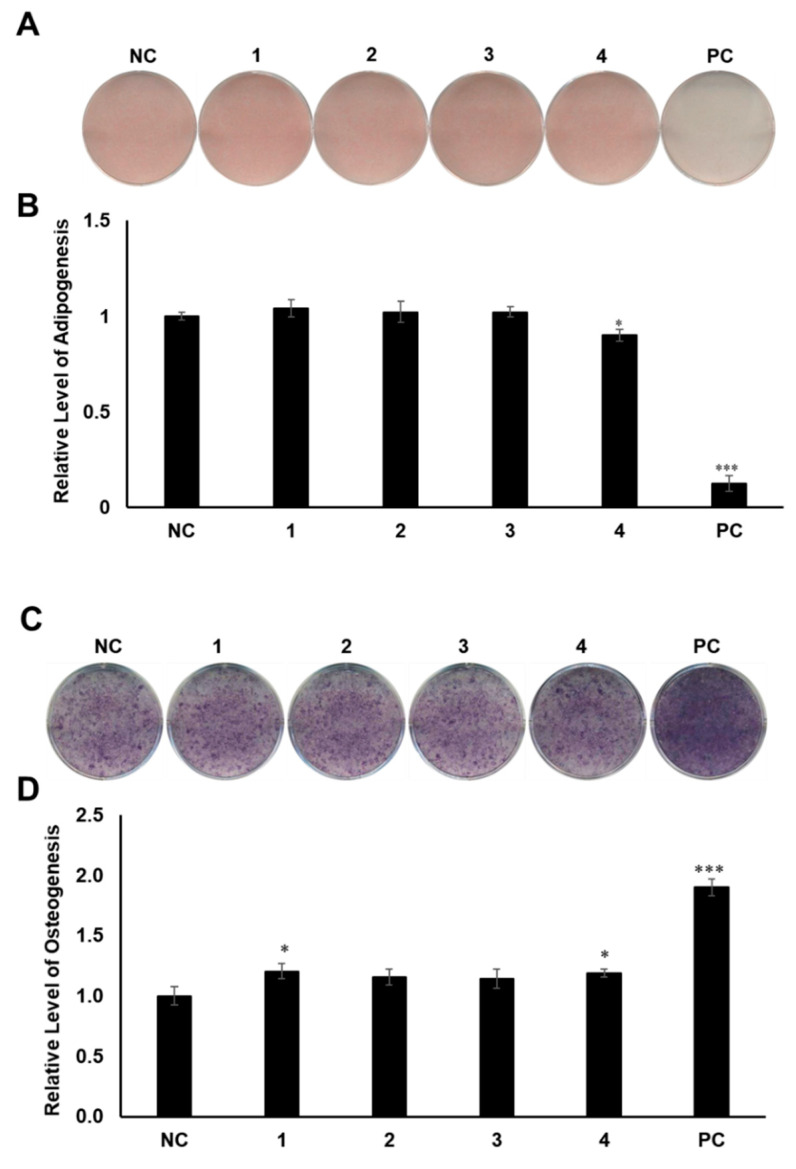
The effects of compounds **1–4** on the differentiation of mesenchymal stem cells (MSCs) toward adipocytes and osteoblasts. (**A**) Suppressive effects of compounds **1–4** on the adipogenic differentiation of MSCs. C3H10T1/2 cells were treated with 20 μM of compounds **1–4**. After adipogenic differentiation, the cells were stained with Oil Red O (ORO). (**B**) The intensity of stained lipid droplets was quantitatively examined. (**C**) Stimulatory effects of compounds **1–4** on osteogenic differentiation of MSCs. Fully differentiated C3H10T1/2 cells were stained with alkaline phosphatase (ALP) on day 9 post osteogenic differentiation with 20 µM compounds **1–4**. (**D**) ALP enzyme activity was determined in osteogenically differentiated C3H10T1/2 cells treated with compounds **1–4**. The values were calculated relatively by setting the untreated negative control (NC) to 1. Resveratrol (20 μM) and oryzativol A (5 μM) were added to the experimental set for adipogenesis and osteogenesis, respectively, as a positive control (PC). * denotes *p* < 0.05 and *** denotes *p* < 0.001.

MSCs are pluripotent cells in bone marrow that are known to differentiate into osteocytes and adipocytes. As microenvironmental changes cause alterations in the regulation of gene expression in MSC differentiation, the alterations of related gene expression might disturb the balance between adipocyte progenitor and osteoprogenitor cells in patients with osteoporosis [27,28,29]. Thus, a therapy that can regulate gene expression in MSCs would be promising for the management of postmenopausal osteoporosis. To determine the regulatory effects of compounds **1–4** on MSC differentiation between adipogenesis and osteogenesis, all the compounds were examined for their effects on the differentiation of murine MSCs into adipocytes or osteoblasts. The murine MSC line C3H10T1/2 was treated with 20 µM of the compounds during adipogenesis, and the differentiated cells were stained with Oil Red O (ORO). Compound **4** slightly reduced the formation of lipid droplets, resulting in fewer ORO-stained cells, compared to the normally differentiated adipocytes (Figure 4A,B). In addition, C3H10T1/2 cells were cultured in osteogenesis-inducing media in the presence of compounds **1–4**. The cells were then stained for alkaline phosphatase (ALP), which is considered a distinctive marker of osteoblast differentiation [30]. Cells treated with compounds **1** and **4** showed slightly higher staining intensity and ALP enzyme activity than the negative control group (Figure 4C,D).

**Figure 5:**
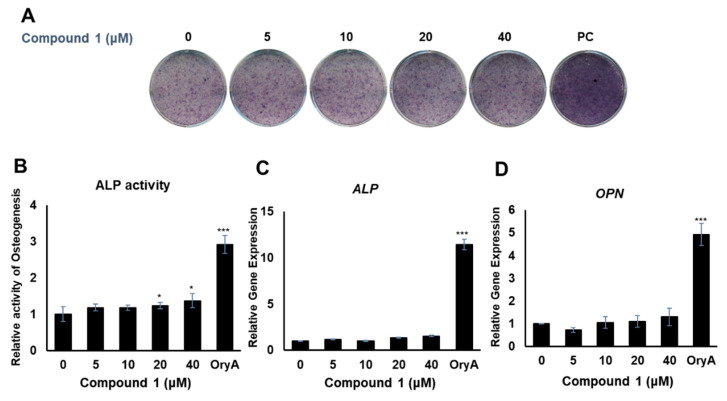
The effects of compound **1** on osteogenic differentiation. C3H10T1/2 cells were treated with sequential concentrations (5, 10, 20, and 40 μM) of compound **1** during osteogenic differentiation. The effects of compound **1** were evaluated through ALP staining (**A**). The cells were evaluated by measuring the ALP activity (**B**). The mRNA expression of *ALP* (**C**) and *OPN* (**D**) was measured by real-time PCR. Oryzativol A (OryA) at a concentration of 5 μM was used as a positive control (PC). * denotes 0.01 < *p* < 0.05 and *** denotes *p* < 0.001.

Compounds **1** and **4** showed the regulatory effects on the differentiation between osteogenesis and adipogenesis of MSCs (Figure 4). Among the active compounds, compound **4** was not sufficient for further experiments to examine its effects. To test the effects of compound **1** on osteogenic differentiation, C3H10T1/2 cells were stained with ALP (Figure 5A), and ALP enzyme activity was measured (Figure 5B). Our results indicated that increased concentrations of compound **1** led to the formation of darker-colored cells (Figure 5A), which indicated that the treated cells exhibited greater promotion of bone differentiation than the control group (Figure 5B). Moreover, compound **1** slightly enhanced the gene expression of *ALP* (Figure 5C) and osteopontin (*OPN*) (Figure 5D), which are osteogenesis-related factors, during osteogenic differentiation in a dose-dependent manner.

## 3. Materials and Methods

### 3.1. Plant Material

Plant material (fruits of *P. multiflorus* var. *albus*) was provided by Dongkook Pharm. Co., Ltd. (Suwon, Korea). The fruits of *P. multiflorus* var. *albus* cultivated in Egypt were purchased from the Weihai Solim trading Co., Ltd. (Beijing, China) in March 2019. The material was authenticated by one of the authors (K.H.K.) and Dongkook Pharm. Co., Ltd. A voucher specimen of the material (DKB117-PM-2018-0814) was deposited at the R&D Center, Dongkook Pharm. Co., Ltd.

### 3.2. Extraction and Isolation

The fruits (1 kg) of *P. multiflorus* var. *albus* were cut into small pieces and then extracted twice with 5-fold volumes of 30% ethanol (*v*/*v*) at 80 °C for 10 h. The extracts were filtered, and the filtrate was concentrated using a rotary evaporator. The resultant extract was fully dried by freeze-drying to obtain the crude ethanolic extract powder (110 g). The extract powder (100 g) was suspended in 700 mL of distilled water and then sequentially partitioned with hexane, MC, EtOAc, and *n*-butanol three times. Four major fractions with different polarities were obtained: hexane-soluble (31 mg), MC-soluble (195 mg), EtOAc-soluble (49 mg), and BuOH-soluble (2.4 g) fractions. The isolation procedure for the compounds was conducted by monitoring via TLC and LC/MS-based analysis. First, the hexane-soluble fraction (31 mg) was applied to Sephadex LH-20 column chromatography and eluted with 100% methanol, yielding two fractions (H_A_ and H_B_). Subfraction H_A_ (14 mg) was purified by semi-preparative HPLC (MeOH/H_2_O, 65:35) to isolate compounds **2** (*t*_R_ 22.4 min, 0.7 mg) and **3** (*t*_R_ 34.8 min, 0.5 mg). However, the isolation of compounds without any impurities failed in the MC-soluble fraction. The EtOAc-soluble fraction (49 mg) was separated directly by semi-preparative reversed HPLC with a gradient solvent system from 30% MeOH/H_2_O to 43% MeOH/H_2_O for 80 min to purify compound **1** (*t*_R_ 37.5 min, 2.3 mg). Finally, the BuOH-soluble fraction (2.4 g) was subjected to reverse-phase MPLC using a gradient solvent system from 5% MeOH/H_2_O to 100% MeOH/H_2_O for 90 min to obtain three fractions (B_A_, B_B_, and B_C_). Subfraction B_A_ (1.1 g) was subjected to reverse-phase MPLC using a different gradient solvent system from 5% MeOH/H_2_O to 30% MeOH/H_2_O for 110 min to produce four subfractions (B_A1_–B_A4_). Subfraction B_A2_ (45 mg) was separated by semi-preparative HPLC with a gradient solvent system from 20% MeOH/H_2_O to 60% MeOH/H_2_O for 70 min to give compound **4** (*t*_R_ 25.5 min, 1.2 mg).

### 3.3. Cell Culture and Differentiation

C3H10T1/2 mouse MSCs were cultured in Dulbecco’s modified Eagle’s medium (DMEM) supplemented with 1% penicillin-streptomycin (P/S) and 10% fetal bovine serum (FBS) at 37 °C in a 5% CO_2_ incubator. For adipogenic differentiation, C3H10T1/2 cells were plated in a 6-well plate (a density of 5 × 10^5^ cells/mL). The cells were then treated with 1 μM dexamethasone, 5 μg/mL insulin, 10 μM troglitazone, and 0.5 mM 3-isobutyl-1 methylxanthine for 48 h. Subsequently, the cells were cultured for an additional 72 h with 5 μg/mL insulin and 10 μM troglitazone. During osteogenesis, 20 µM of compounds **1–4** was added to the cells, and resveratrol (20 µM) was used as a positive control. For osteogenic differentiation, C3H10T1/2 cells were exposed to DMEM (5% FBS, 1% P/S) containing 50 μg/mL ascorbic acid and 10 mM *β*-glycerophosphate for 9 days. During osteogenic differentiation, 20 µM of compounds **1–4** was added to the cells, and 5 µM oryzativol A was used as a positive control.

### 3.4. Oil Red O Staining

Cultured cells were washed with phosphate-buffered saline and fixed in 10% neutral-buffered formalin at room temperature for 1 h. The cells were then stained with 0.5% filtered ORO stock solution (Sigma, Saint Louis , MO, USA) in isopropanol. To evaluate the intracellular triglyceride content, the stained cells were redissolved with isopropanol. The absorbance at a wavelength of 520 nm was measured.

### 3.5. Alkaline Phosphatase Staining

Cultured cells were washed with 2 mM MgCl_2_ and then incubated with ALP buffer (100 mM Tris-HCl, pH 9.5; 100 mM NaCl, 0.05% Tween-20, and 10 mM MgCl_2_). The cells were then incubated in an ALP buffer containing 0.2 g/mL of 5-bromo-4-chloro-3-indolyl phosphate (Sigma, USA) and 0.4 mg/mL of nitro-blue tetrazolium (Sigma, USA). After washing with 0.5 mM of ethylenediaminetetraacetic acid, the cells were fixed by 10% neutral-buffered formalin.

### 3.6. mRNA Isolation and Real-Time PCR

RNA was isolated from the cells via NucleoZOL reagent (NucleoZOL; Macherey-Nagel GmbH & Co., KG, Dylan, Germany). Then, complementary DNA (cDNA) was synthesized from total RNA (0.5 μg) by a ReverTraAce qPCR RT Master Mix Kit (FSQ-201; Toyobo, Osaka, Japan) with random primers. The synthesized cDNA was mixed with the amplification mixture including the Thunderbird SYBR qPCR Mix (Toyobo) and primers. The cDNA was then subjected to 40 PCR amplification cycles by a Thermal Cycler Dice (Takara, Kusatsu City, Japan). The results were normalized to the expression of 36b4. The primers used in this study are as follows: 

*Acidic ribosomal phosphoprotein P0 (36b4): forward 5′-AGATGCAGCAGATCCGCAT-3′, reverse 5′-GTTCTTGCCCATCAGCACC-3′; ALP: forward 5′-CCATTCTGGCCCACCAAC-3′, reverse 5′-AATGCGAGTGGTCTTCCATCA-3′; osteopontin (OPN): forward 5′-CTGGCAGCTCAGAGGAGAAG -3′, reverse 5′- CAGCATTCTGTGGCGCAAG-3′.*

### 3.7. Statistical Analysis

Each sample was tested in triplicate, and the test was repeated three times. Data are expressed as the mean ± standard deviation (SD). Differences between the control and experimental groups were analyzed via a two-tailed unpaired Student’s *t*-test, and statistical significance was defined as *p* < 0.05.

## 4. Conclusions

In the present study, the phytochemical exploration of *P. multiflorus* var. *albus* fruits resulted in the isolation of four compounds: denatonium (**1**), *trans*-ferulic acid ethyl ester (**2**), eugenin (**3**), and α-L-glutamyl-L-Leucine (**4**). The chemical structures of isolates **1–4** were elucidated by 1D and 2D NMR, HR-ESIMS, and LC/MS analyses. Intriguingly, to the best of our knowledge, denatonium (**1**), which is known to be the most bitter chemical compound, was identified from natural sources for the first time, and compounds **2–4** were reported for the first time from *P. multiflorus* var. *albus* in this study; however, compound **2** turned out to be an artifact produced by the extraction with ethanol. Compound **4** exhibited the dual functions of inhibiting adipogenesis and promoting osteogenesis, showing regulatory effects on MSC differentiation. Although the stimulatory effect of the active compounds on osteogenic differentiation was far behind that of the positive control, oryzativol A and compounds **1** and **4** apparently helped promote the differentiation of MSCs into osteocytes.

## Figures and Tables

**Table 1:** ^1^H and ^13^C NMR data of compound **1** in CD_3_OD (δ in ppm, 850 MHz and 212.5 MHz for ^1^H and ^13^C, respectively) ^a^.

Position	Denatonium (1)	
	*δ*_H_ (*J* in Hz)	*δ*_C_
1		128.7 C
2	7.64, d (7.5)	134.1 CH
3	7.58, t (7.5)	130.7 CH
4	7.62, t (7.5)	132.3 CH
5	7.58, t (7.5)	130.7 CH
6	7.64, d (7.5)	134.1 CH
7	4.94, s	63.4 CH_2_
8	4.16, s	55.7 CH_2_
9		164.1 C
10		134.2 C
11		136.7 C
12	7.17, d (7.0)	129.5 CH
13	7.18, t (7.0)	129.1 CH
14	7.17, d (7.0)	129.5 CH
15		136.7 C
16	2.30, s	18.7 CH_3_
17	2.30, s	18.7 CH_3_
1′	3.67, m	54.9 CH_2_
2′	1.56, t (7.5)	8.4 CH_3_
1″	3.67, m	54.9 CH_2_
2″	1.56, t (7.5)	8.4 CH_3_

^a^ Coupling constants (Hz) are given in parentheses. ^13^C NMR assignments were based on HSQC, ^1^H-^1^H COSY, and HMBC experiments.

## Supplementary Materials

1D and 2D NMR spectra of denatonium (**1**) and general experimental procedures are available online at https://www.mdpi.com/article/10.3390/plants10102205/s1, Figure S1: HR-ESIMS data of **1**, Figure S2: ^1^H NMR spectrum of **1** (CD_3_OD, 850 MHz), Figure S3: ^13^C NMR spectrum of **1** (CD_3_OD, 212.5 MHz), Figure S4: ^1^H-^1^H COSY spectrum of **1** (CD_3_OD), Figure S5: HSQC spectrum of **1** (CD_3_OD), Figure S6: HMBC spectrum of **1** (CD_3_OD), Figure S7: The total ion chromatogram of ethanol and methanol that we used for extraction in positive ion mode by UPLC-QTOF MS.
