# Supplementary material for: Phytochemical Investigation of Bioactive Compounds from White Kidney Beans (Fruits of Phaseolus multiflorus var. Albus): Identification of Denatonium with Osteogenesis-Inducing Effect
Source: Plants (Basel). 2021 Oct 17;10(10):2205. doi: 10.3390/plants10102205 (PMC8540745; doi:10.3390/plants10102205)
Supplement: Supplementary file 1 [file plants-10-02205-s001.zip › plants-1418614-supplementary.pdf]

Supplementary Data

Phytochemical Investigation of Bioactive Compounds from White Kidney Beans (Fruits of *Phaseolus multiflorus* var. *albus*): Identification of Denatonium with Osteogenesis-Inducing Effect

Yong Hoon Lee [1,†], Joo-Hyun Hong [1,†], Kun Hee Park [2], Seon-Hee Kim [3], Jin-Chul Kim [4], Do Hoon Kim [5],
Yu Hwa Park [5], Kye Wan Lee [5], Jung Kyu Kim [6,*] and Ki Hyun Kim [1,*]

[1] School of Pharmacy, Sungkyunkwan University, Suwon 16419, Korea; yhl2090@naver.com (Y.H.L.); ehong@skku.edu (J.-H.H.)
[2] Department of Food Science and Biotechnology, Sungkyunkwan University, Suwon 16419, Korea; soske@skku.edu
[3] Sungkyun Biotech Co., Ltd., Suwon 16419, Korea; seonhee31@gmail.com
[4] Natural Product Informatics Research Center, KIST Gangneung Institute of Natural Products, Gangneung 25451, Korea; jckim@kist.re.kr
[5] R&D Center, Dongkook Pharm. Co., Ltd., Suwon 16229, Korea; kdh2@dkpharm.co.kr (D.H.K.); pyh@dkpharm.co.kr (Y.H.P.); lkw1@dkpharm.co.kr (K.W.L.)
[6] School of Chemical Engineering, Sungkyunkwan University, Suwon 16419, Korea
* Correspondence: legkim@skku.edu (J.K.K.); Tel.: +82-31-290-7254; khkim83@skku.edu (K.H.K.); Tel.: +82-31-290-7700
† These authors contributed equally to this study.

Figure S1 : HR-ESIMS data of **1**

Figure S2 : ^{1}H NMR spectrum of **1** (CD_3OD, 850 MHz)

Figure S3 : ^{13}C NMR spectrum of **1** (CD_3OD, 212.5 MHz)

Figure S4 : ^{1}H-^{1}H COSY spectrum of **1** (CD_3OD)

Figure S5 : HSQC spectrum of **1** (CD_3OD)

Figure S6 : HMBC spectrum of **1** (CD_3OD)

General experimental procedures

Figure S7 : The total ion chromatogram of ethanol and methanol that we used for extraction in positive ion mode by UPLC-QTOF MS.

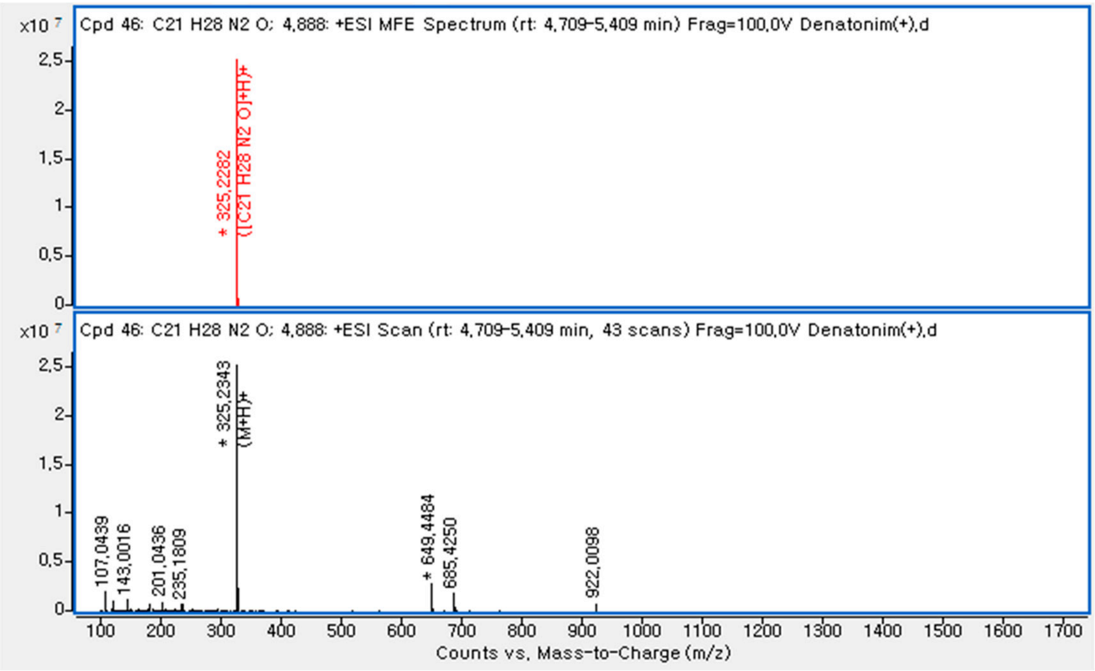

Figure S1 : HR-ESIMS data of **1**

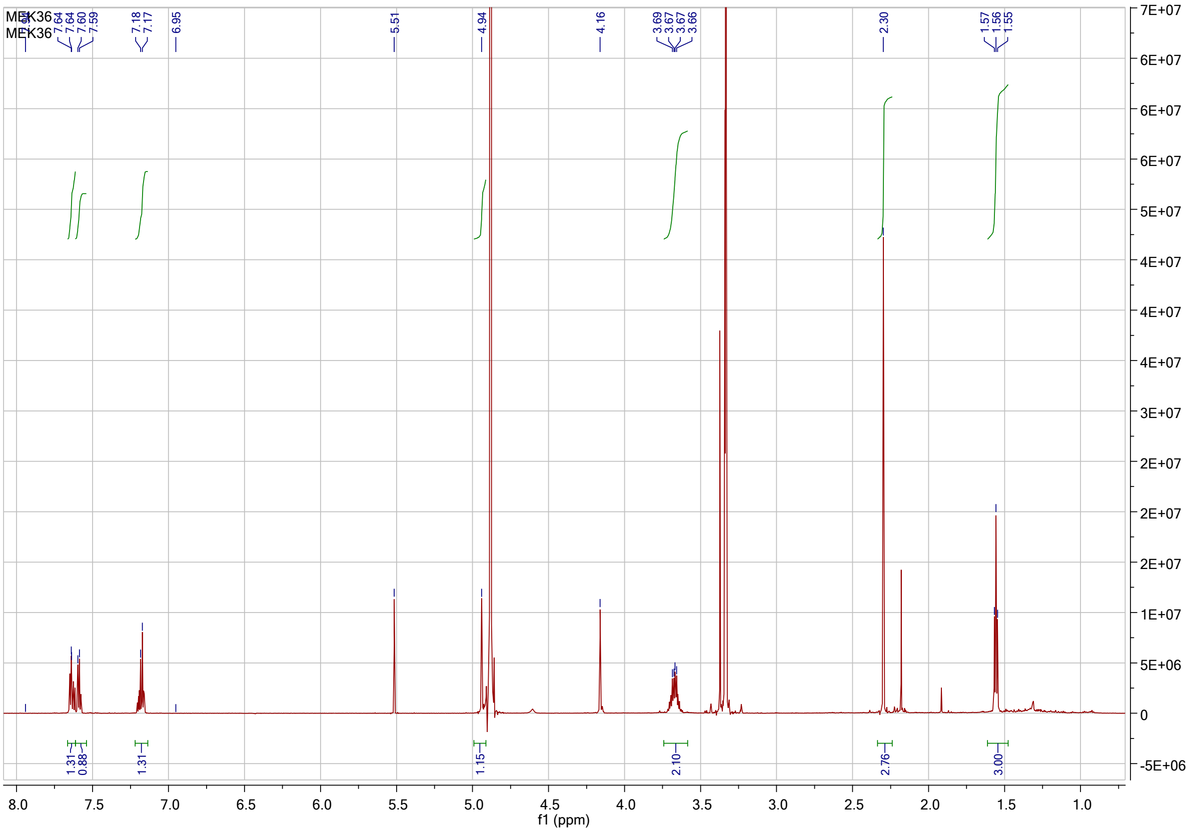

Figure S2 : ^{1}H NMR spectrum of **1** (CD_3OD, 850 MHz)

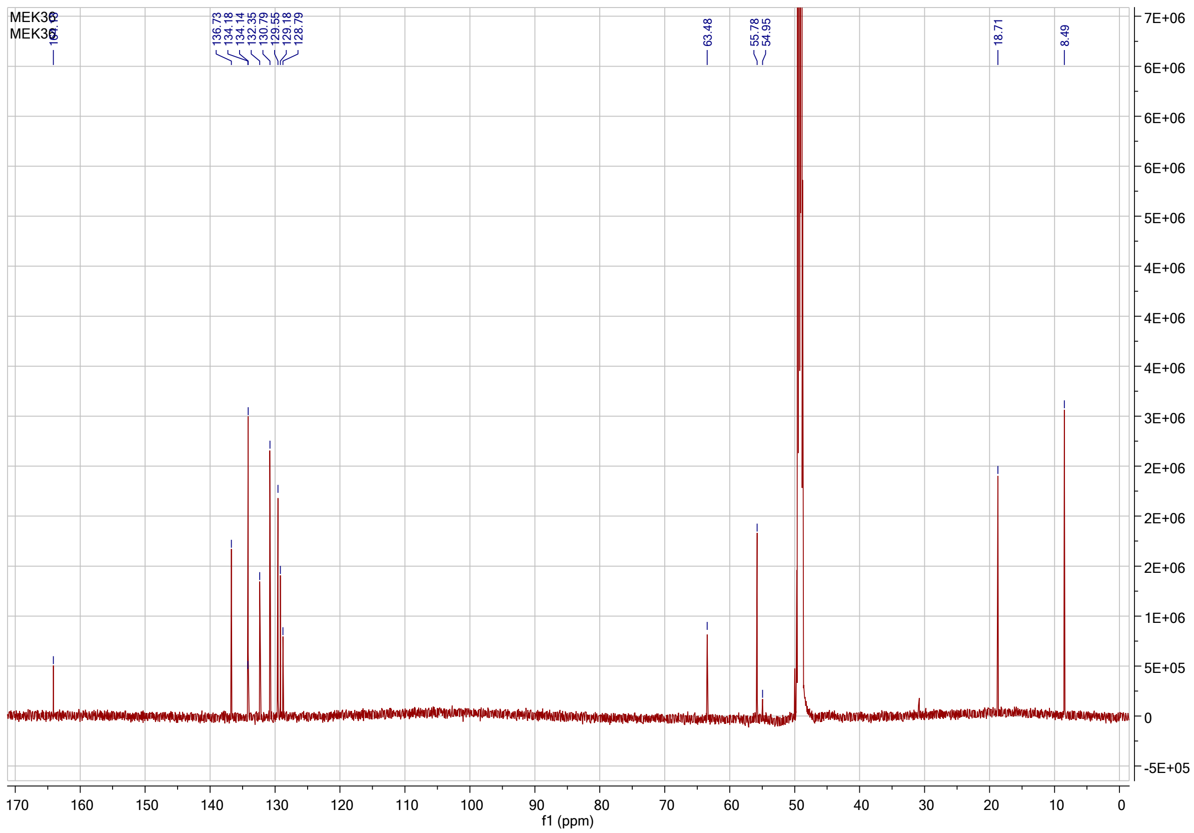

Figure S3 : ^{13}C NMR spectrum of **1** (CD_3OD, 212.5 MHz)

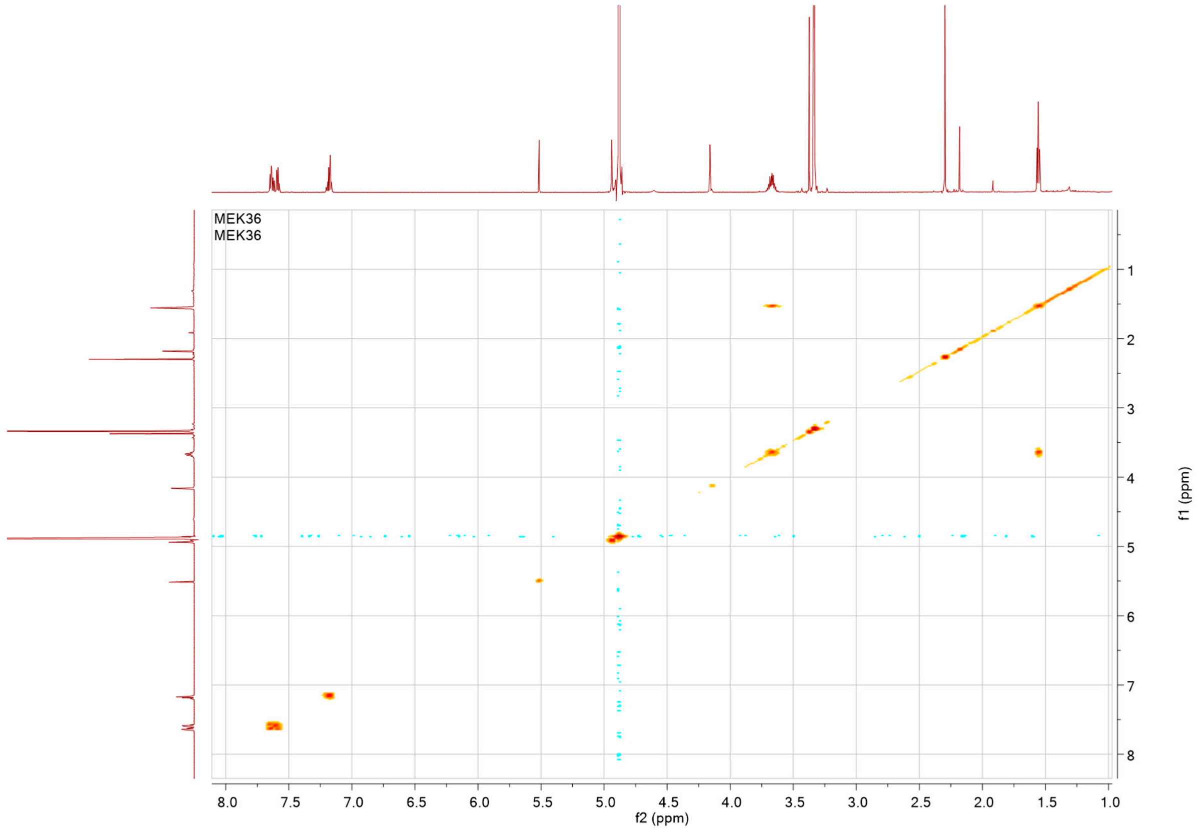

Figure S4 : ^{1}H-^{1}H COSY spectrum of **1** (CD_3OD)

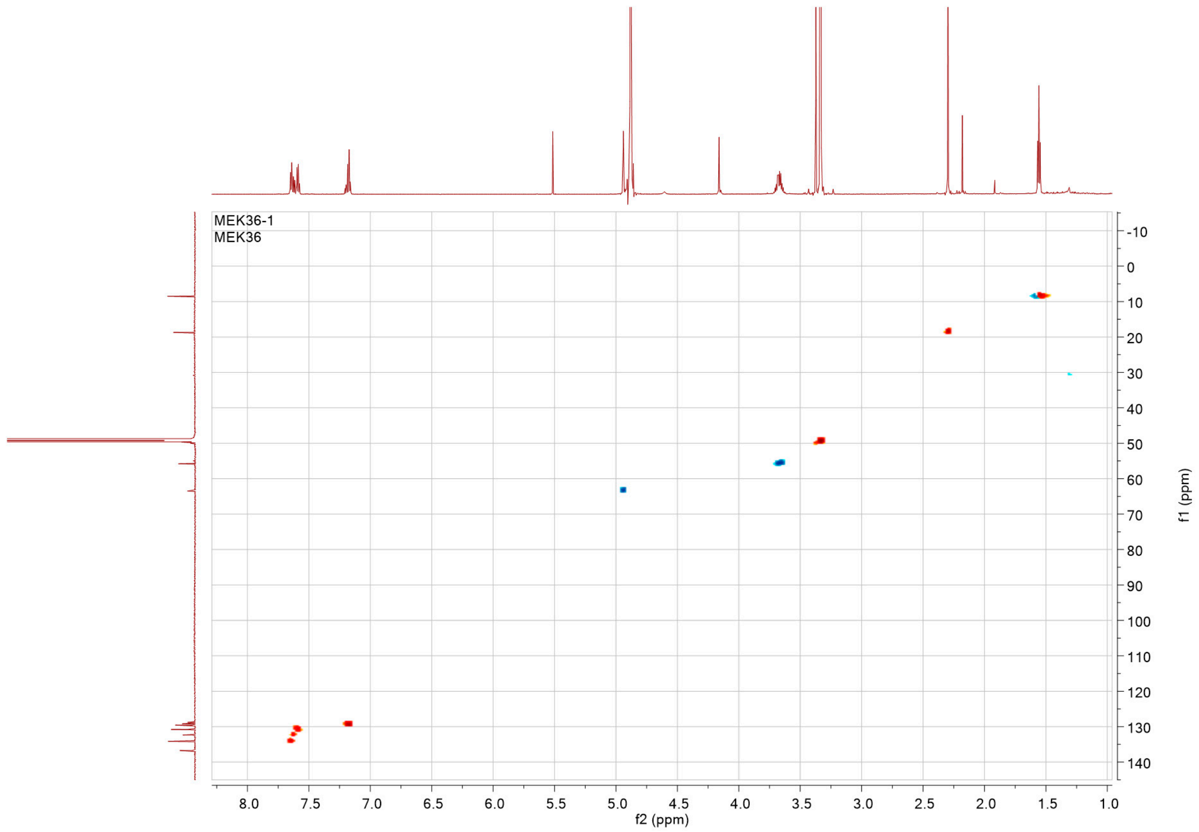

Figure S5 : HSQC spectrum of **1** (CD_3OD)

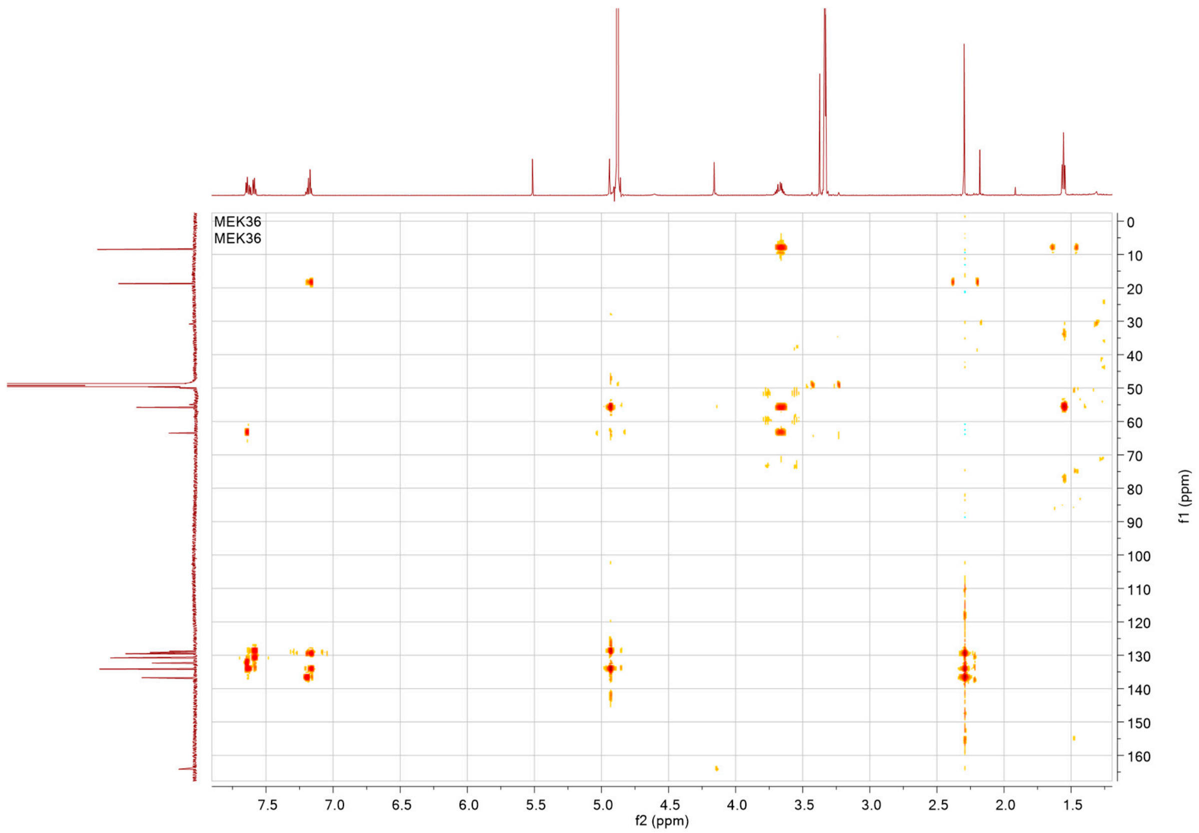

Figure S6 : HMBC spectrum of **1** (CD_3OD)

General experimental procedures

Optical rotations were acquired using a JASCO P-2000 polarimeter (JASCO, Easton, MD, USA). Ultraviolet (UV) spectra were recorded on an Agilent 8453 UV-visible spectrophotometer (Agilent Technologies, Santa Clara, CA, USA). NMR spectra were recorded using a Bruker AVANCE III HD 850 NMR spectrometer with a 5-mm TCI CryoProbe, operated at 850 MHz (^{1}H) and 212.5 MHz (^{13}C). The chemical shifts are represented in ppm (δ) for the ^{1}H and ^{13}C NMR analyses. HRESIMS spectra were recorded on an Agilent 1290 Infinity II series with a 6545 LC/Q-TOF mass spectrometer (Agilent Technologies) with an Agilent EclipsePlus C_{18} column (2.1 mm × 50 mm i.d., 1.8 µm; flow rate: 0.3 mL/min) maintained at 20 °C. Medium-pressure liquid chromatography (MPLC) was performed on a Smart Flash AKROS (Yamazen, Osaka, Japan) using an analytical Universal ODS-SM 120 Å column (3.0 × 20.0 cm, 50 µm) (Yamazen) and Universal Premium Silica gel 60 Å column (2.3 × 12.3 cm, 30 µm) (Yamazen). Semi-preparative HPLC was performed on a Waters 1525 binary HPLC pump with a Waters 996 photodiode array detector (Waters Corporation, Milford, CT, USA) using a Phenomenex Luna Phenyl-hexyl 100 Å column (250 × 10 mm, 10 µm; flow rate: 2 mL/min). The LC/MS analysis was performed on an Agilent 1200 series HPLC system with a diode array detector and a 6130 Series ESI mass spectrometer using an analytical Kinetex C_{18} 100 Å column (100 mm × 2.1 mm i.d., 5 µm; flow rate: 0.3 mL/min) (Phenomenex). Silica gel 60 (230–400 mesh; Merck, Darmstadt, Germany), RP-C_{18} silica gel (230–400 mesh; Merck), and silica Sep-Pak Vac 6 cc cartridges (Waters) were used for column chromatography. Sephadex LH-20 (Pharmacia, Uppsala, Sweden) was used as the packing material for molecular sieve column chromatography. TLC was performed using precoated silica gel F_{254} plates and RP-C_{18} F_{254s} plates (Merck), and spots were detected under UV light or by heating after spraying with anisaldehyde-sulfuric acid.

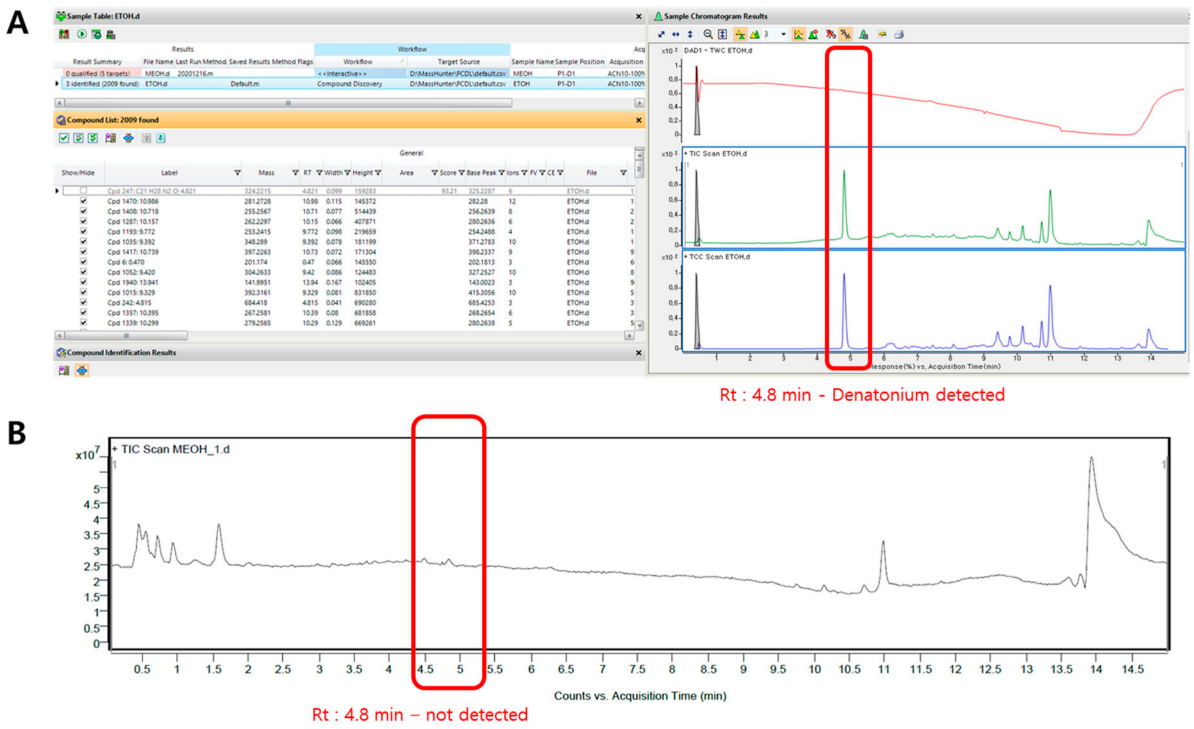

Figure S7 : The total ion chromatogram of ethanol (A) and methanol (B) that we used for extraction in positive ion mode by UPLC-QTOF MS.

Method: The analysis was performed on an Agilent 1290 Infinity II HPLC instrument (Agilent Technologies, Foster City, CA, USA) coupled to a G6545B quadrupole time-of-flight (Q-TOF) mass spectrometer (Agilent Technologies). The analysis was carried out on an Agilent EclipsePlus C18 column (2.1 mm × 50 mm, 1.8 µm) maintained at 20 °C with flow rate of 0.3 mL/min. The mobile phrase consisted of solvent A (water + 0.1% formic acid) and solvent B (100% acetonitrile). The gradient elution system was eluted with 90% A → 100% B (0-10 min), 100% B (11–16 min), and 90% A (16–20 min) for equilibration before the next injection. The samples were monitored at 254 and 210 nm for chromatographic run. Mass spectral analysis was carried out on a MassHunter software (Agilent).
